# Response processes for patients providing quantitative self-report data: a qualitative study

**DOI:** 10.1007/s11136-024-03749-2

**Published:** 2024-08-14

**Authors:** Øyvind Grindheim, Andrew McAleavey, Valentina Iversen, Christian Moltu, Kristin Tømmervik, Hege Govasmark, Heidi Brattland

**Affiliations:** 1 Department of Mental Health, Norwegian University of Science and Technology, Trondheim, Norway; 2 District General Hospital of Førde, Førde, Norway; 3 Department of Health and Caring Sciences, Western Norway University of Applied Science, Førde, Norway; 4 Weill Cornell Medical College, New York, NY USA; 5 Clinic of Substance Use and Addiction Medicine, St. Olavs University Hospital, Trondheim, Norway; 6 Department of Psychology, Norwegian University of Science and Technology, Trondheim, Norway; 7 Nidelv Mental Health Center, St. Olavs University Hospital, Trondheim, Norway

**Keywords:** Response process, Self-report measure, Routine outcome monitoring, Clinical feedback, Qualitative research, Cognitive interviewing

## Abstract

**Objective:**

To identify factors that influence response processes for patients providing quantitative self-report data. Secondly, due to the lack of integrative and explanatory models in this area, to develop a model of patients’ response processes that can guide what to look for when considering validity evidence and interpreting scores on individual items.

**Methods:**

Participants (*n* = 13) were recruited from a specialized substance use disorder treatment clinic and interviewed while responding to items from a clinical feedback system implemented for routine outcome monitoring in that setting. The interview approach was based on cognitive interviewing. Data collection and analysis were inspired by a grounded theory approach.

**Results:**

We identified several variables that influenced the participants’ response processes. The variables were organized into five categories: context-related variables; item-related variables; response base variables; reasoning strategies; and response selection strategies. We also found that the participants’ responses for many items were affected by different aspects of the response process in ways that are relevant to interpretation but not necessarily discernible from the numerical scores alone, and we developed response categories to capture this.

**Conclusion:**

The findings suggest that patients providing quantitative self-report data encounter conditions in the response process that challenge and influence their ability to convey meaning and accuracy. This results in responses that for many of the items reflect messages important for interpretation and follow-up, even if it does not appear from the numerical scores alone. The proposed model may be a useful tool when developing items, assessing validity, and interpreting responses.

**Supplementary Information:**

The online version contains supplementary material available at 10.1007/s11136-024-03749-2.

## Plain English summary

Self-report measures are frequently used within many clinical settings to plan, adapt, and evaluate treatments. In many cases self-report can be an effective mean to obtain valuable information that can improve the individual patient’s treatment. However, the quality of the information provided is uncertain for many self-report measures used today, and patients report that the questions may be ambiguous and difficult to answer. A central reason for this may be that previous research on self-report measures has tended to overlook an important aspect of measurement, namely the patients’ response processes, i.e. what they do, think, and feel, when providing quantitative self-report data. Without such knowledge it is difficult to know exactly what the self-report really measures. The aim of this study was therefore to increase the understanding of response processes for patients providing quantitative self-report data. In the study, we interviewed a sample from a substance use disorder treatment setting while they were responding to items from a self-report measure. Based on the interviews we investigated and analyzed the psychological processes that occurred between reading the question and providing an answer. The findings focus on what constitutes and influences the response process and outcome. The knowledge gained from this study can be used to develop better self-report measures and improve the interpretations based on the results of such measures.

## Introduction

Quantitative self-report measures should have strong validity evidence to support their use in a given clinical setting, but this is not always the case [1, 2]. The Standards for Educational and Psychological Testing defined five sources of validity evidence: content-related, internal structure, relationship with other variables, response processes, and consequences [3]. A review by Zumbo and Chan [2] documented, however, that previous research has largely overlooked response processes as a source of validity evidence. According to Hubley and Zumbo [4] response processes can be defined as *the mechanisms that underlie what people do, think, or feel when interacting with, and responding to the item or task, and are responsible for generating observed test score variation (p.2)*. Without knowledge about these underlying mechanisms, it is difficult to know what kind of impact the measures may have on the responders, and whether different numeric outcomes from items reflect the intended real-world differences or if they are the results of ideographic reasoning processes. Even if other evidence suggests validity, response process data may reveal hidden weaknesses.

In recent years, effect studies have demonstrated that routine outcome monitoring with clinical feedback systems (ROM/CFS) is a helpful adjunct to psychological treatments for a wide range of disorders and in several treatment settings [5]. Ideally, ROM/CFS involves three sequential phases: 1) collecting treatment-relevant patient data via self-report measures regularly throughout the treatment course; 2) feeding this information back to the therapist(s), and sometimes also to the patient; and 3) adapting treatment according to the patient’s reported needs [6].

Response processes may be particularly important when it comes to clinical feedback systems because such systems can be considered both assessment instruments and clinical interventions. A recent review of qualitative studies documented that feedback systems are used clinically in multiple ways by both psychotherapists and patients, including intra- and interpersonal purposes, as well as assessment [7]. This highlights that in a clinical setting, a patient’s response is not only a measurement act but also a potential communication between the patient and treatment provider [8]. Consequently, the evaluation of the response process related to clinical feedback systems must consider not only its ability to measure what it is meant to measure but also how patients experience completing these questionnaires, and what they consider when selecting responses.

The most extensive research related to question-answering processes on self-report measures thus far comes from studies investigating cognitive aspects of survey measures [9–15]; see also [8]. While this research provides valuable contributions concerning cognitive aspects influencing the accuracy of the responses, it tends to focus on survey measures addressing other areas (e.g. memory of events, behavior, or attitudes) than the self-evaluative items in ROM/CFS measures. In addition, because the conceptualization of components in the response process is typically based on established concepts from cognitive sciences in these studies, they do not necessarily reflect the response process as seen from the perspective of responders and in a treatment context.

To understand the underlying mechanisms of what people do, think, or feel when providing quantitative self-report such as in ROM/CFS, we may need to limit preconceptions and instead build conceptual knowledge bottom-up through induction and abduction, i.e. develop concepts and hypothesis about relationships grounded in empirical data [16]. In other words, we need to first study what people do, think, and feel before inferring underlying mechanisms that are responsible for generating observed test score variations.

In this study, we interviewed a sample of patients in a substance use disorder treatment clinic while they were responding to items from a clinical feedback system already implemented in that setting. The study aimed to identify factors that constitute and influence the patients’ response processes and explore how these response processes might influence test scores. In addition, due to the lack of existing integrative and explanatory models of patients’ response processes, we wanted to use our findings to develop a model that can guide what to look for when considering validity evidence and interpreting scores on individual items in quantitative self-report measures.

## Methods

### Methodological approach

Data collection and analyses in this study were structured to allow for a new theory to emerge. The interview approach was based on cognitive interviewing [17, 18] which is an approach to interviewing characterized by collecting additional verbal information about the response process while administering a self-report measure or test [18]. To identify factors that influence the response process and to develop an integrative model of the process across different items and responders, we used elements from a grounded theory approach in the analysis, including simultaneous data collection and analysis, constant comparison of data, and emphasis on developing new concepts and theory grounded in data [19–21].

### Participants and recruitment

The participants (*n* = 13) were recruited from a specialized substance use disorder treatment clinic. Participant characteristics are summarized in Table 1 (See Supplementary Information 2).

**Table 1:** Participant characteristics (*n* = 13)

	*n (%)*	*R*	*M (SD)*
Age		22–72	48.15 (14.8)
Female	8 (62%)		
Male	5 (38%)		
Treatment setting			
Outpatient	10 (77%)		
Inpatient	3 (23%)		
Employment status			
Employed	6 (46%)		
Disability benefits/Retired	7 (54%)		
Types of substance use			
Alcohol	8 (62%)		
Cocaine	2 (16%)		
Opioids	1 (8%)		
Sedatives	1 (8%)		
Cannabis	1 (8%)		
Amphetamine	1 (8%)		
Unspecified	1 (8%)		
Duration of substance use disorder (years)		0–45	12.15 (15.01)
Comorbid mental health disorders			
Mood disorders	4		
Anxiety disorders	2		
Post-traumatic stress disorder	2		
Eating disorder	1		
Experience with using NF (months)		0–18	6.15 (5.32)

### Materials

Norse Feedback (NF) [22] is a clinical feedback system implemented in several different treatment settings. NF consists of over 100 items, with a stem focused on the patient’s self-evaluation over the past week, which are rated on a seven-point Likert scale [23]. The feedback system is administered via mobile technology outside of treatment, resulting in a report that can be reviewed by clinicians before planned clinical encounters. NF covers several treatment-relevant domains (symptoms, alliance, maintaining factors, risk factors, resources, etc.) organized into 20 scales. At the start of a new course of treatment the NF screens for all the domains. In subsequent sessions, the number of items administered is reduced and adapted to the individual patients’ responses via computer adaptive algorithms, after which less relevant scales are collapsed and only represented by a trigger item.

Because the full NF includes over 100 items, only some of which are administered at each assessment occasion, we selected a subset of NF items for the interviews. The items were selected to cover a wide scope and included items targeting symptoms, such as anxiety, depression, suicidality, substance use; therapeutic alliance and needs; sustaining factors such as hopelessness, inner avoidance, situational avoidance; and resources, such as readiness for change, social safety. A minimum of 15 items were administered in each interview. In many of the interviews, however, additional items were included to follow up and clarify issues that needed to be investigated further.

### Data collection and analysis

In the interview, participants were encouraged to imagine that they were responding to these items as part of their treatment. They were presented with the items one by one and encouraged to describe their thought processes while responding. When appropriate, we asked probing questions to elaborate on their internal response process related to each item. All interviews were audio recorded and transcribed verbatim before analysis (See Supplementary Information 1).

In the analysis, all the data material was subjected to an open line-by-line coding to identify meaning units [24]. QSR International NVivo software (version 1.7, released September 2022) was used to organize codes. The initial coding was followed by selective coding with a specific focus on the research question and what seemed to be the participants’ main concerns. New data was incorporated by continuously comparing it to the existing material and factors that constituted and influenced the response process were gradually identified based on patterns in the meaning units. Later in the analysis, the different factors were categorized based on their function in the response process. Finally, the material was subjected to theoretical coding focusing on the relationships between the different categories and how the overall response process influenced the participants’ numerical ratings on specific items.

To promote reflexivity, at the end of each interview the interviewer summarized and reviewed tentative findings together with the participant to check shared understanding and clarify ambiguities. Additionally, short notes (memos) were written to record potential analytic ideas during both data collection and analysis. These memos were kept in a document separate from the rest of the data material and integrated before the final writing up. In keeping with findings from previous research on cognitive aspects in survey measures, we expected the findings to be related to interpretation, retrieval, judgment, and decision processes, but we had no clear idea of how the response process would appear from the responders’ point of view, with self-evaluative items, and in a treatment context.

The first author conducted the interviews and coded the material. After the second round of data collection, tentative categories and conceptualized relationships were discussed in analytic meetings at which ØG, VI, AM, CM, and HB attended. Then the last interviews were conducted for theoretical saturation [25]. All co-authors read through the manuscript and gave feedback on its clarity. The quotes were chosen and initially translated by the first author and then crosschecked by the other authors.

## Results

A main concern for the participants was to find answers that would be accurate but also meaningful in the treatment process. However, in the analysis we found several variables influencing both the response process and challenging the participants efforts to convey meaning and accuracy. We also found that different aspects of the response process were important for how many of the item responses should be interpreted and followed up, and we developed response categories to conceptualize this.

**Fig. 1:**
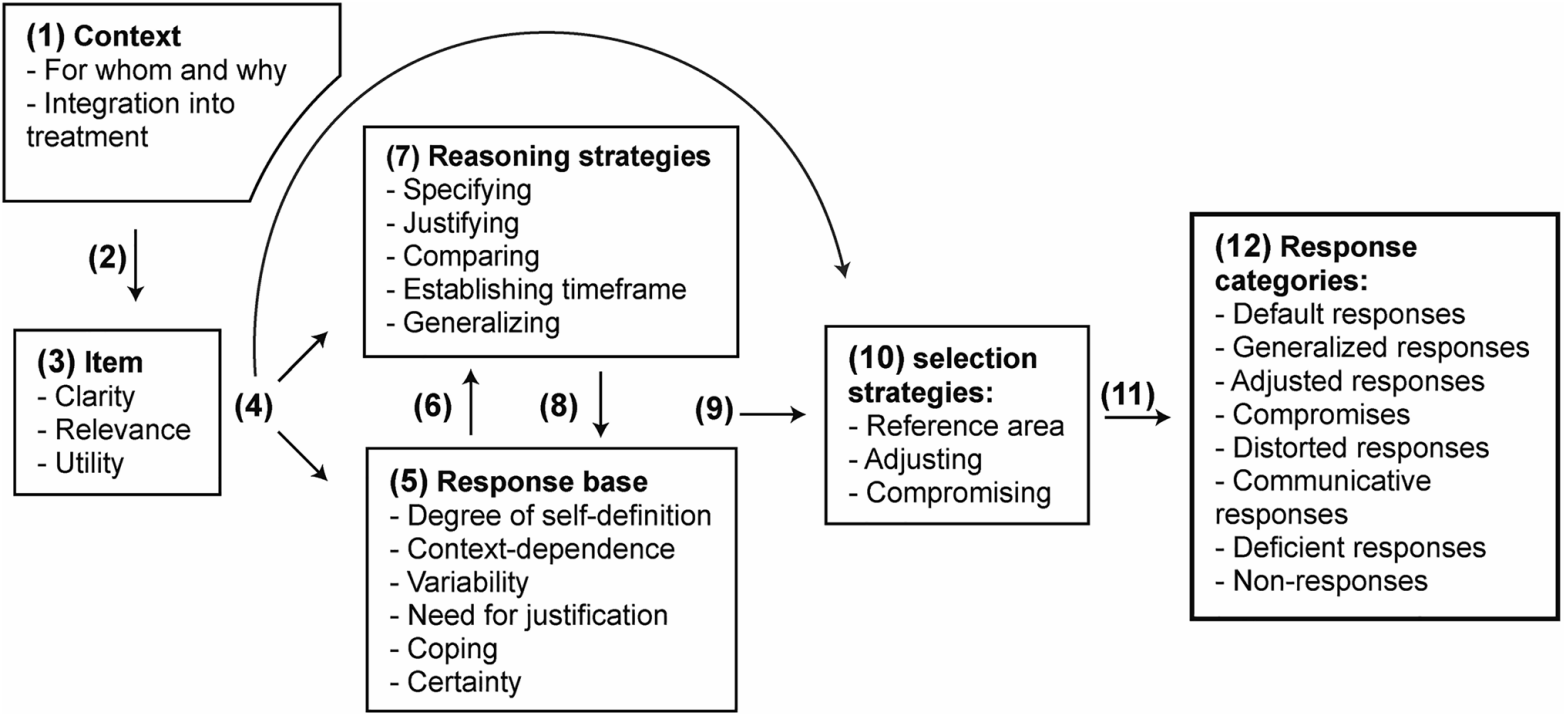
Illustration of the response process categories, variables, and conceptualized relationship. (1) Context-related variables influence (2) the responder’s overall experienced purpose and motivation, before (3) item-related variables impact and trigger (4) different reasoning pathways. (5) Response base variables entail different (6) reasoning requirements. (7) Reasoning strategies are utilized to (8) improve the response base. When the (9) response base is considered sufficient or further reasoning unhelpful, (10) response selection strategies are employed to (11) translate the response into numerical ratings. (12) Due to the influence of different response process variables, the numerical response on many of the items can be categorized based on how they are affected by different aspect of the response process

In the following, we describe how the participants made their way forward to find answers to the different items. Variables that influenced the response process are marked in **bold** and organized into five categories: context-related variables; item-related variables; response base variables; reasoning strategies; and response selection strategies. To build a coherent model we also describe the relationship between the different categories, before ending up with the resulting response categories. Figure 1 provides a visual overview of the categories, variables, and relationships between them. Table 2 provides a more detailed description of the different variables, the response categories, and additional participants’ quotes.

**Table 2:** Summary of response process variables and response categories with additional participant quotes

Response process variables and response categories	Participant quotes
1. **Context-related variables**	
** 1.1. For whom and why** The participants reported different perceptions of who they responded to, and why. Especially in the initial phase of treatment, it was sometimes unclear who the recipient, if any, would be. Some participants said that this might have been explained to them but forgotten. Most participants eventually got the impression that it was for the therapist. It was also typically seen as a therapist’s tool, in the sense that the patient’s responses would make it possible to assess their needs and adapt treatment. A few reported that they responded mostly for their own part and had no clear perception of any recipient in mind, however, still thought that their responses were somehow used to assess and evaluate	“*I think I’ve used this measure to consider how I myself feel I’m doing, more than to think that it will be used or read or used in treatment”* (P1) * “In the beginning, I didn’t quite understand ‘where does this thing go?’ Does it go to someone outside [of treatment] or does it go to the therapist? But gradually I realized that it was for the therapist, and so that we would have something to work on in the session”* (P10) * “P: I think it’s for someone… a person who wants to know the answers to these things* *I: Do you think of someone specific?* *P: No, I don’t. I imagine a stranger”* (P6) * “I guess she [the therapist] used it more for her own part, to get to know me better”* (P2)
** 1.2. Integration into treatment** The participants reported different experiences of how the ROM/CFS system was integrated into treatment. For some the measures were used very actively, meaning that the therapist reviewed the responses and addressed them in the session. Others did not experience any obvious connection between the ROM/CFS responses and what happened in the treatment	“*I don’t know how relevant it feels in treatment. No, not so far, at least. It’s not like something they’ve asked me about*” (P6) “*When I went to [the therapist] it was it was kind of part of the ‘deal’. And she used it very actively, so I got a lot of feedback from her”* (P2) “*My therapist compared it [current responses] to what I responded last time. If the scores were all the same or if there were any differences”* (P9) *“I notice that my therapist is very good at reviewing it [the patient’s responses]. So she has it ready each time, or she looks at it while I am there […] then she says ‘I see how you’ve been feeling this week”* (P4)
**2. Item-related variables**	
** 2.1. Clarity** Encountering the different items, the participants tried to figure out their intended meaning. What was the item about? The initial reactions indicated that it could be unclear for the participants what specific phenomena the items referred to or how the phenomena in question should be interpreted or operationalized. Unclarities were also related to some of the specific terms used in the items. For instance, many participants found words such as “treatment” and “functioning” to be too general	“*It is not clear what they want to know from me*” (P10) *“I don’t understand that one. What is the definition of techniques and exercises?”* (P13) *“But it’s a big word [treatment]. I don’t know. Because it is so much, it’s so many things”* (P3) *“I don’t know what is meant by “I am trying hard to avoid”. I don’t know what that means”* (P12)
** 2.2. Relevance and utility** This category refers to the experienced personal significance of each item. The participants reported a sense of each item’s relevancy and utility for the treatment process or themselves in general. Some items were particularly noticed because they reflected something fundamental or something the participants were working on. Other items were seen as important reminders or items that potentially could uncover risk	*“I think that one is a very good question because it’s like one of those core issues that I have”* (P13) *“The question is a good reminder […] the problem is so fundamental and long-lasting that I cannot simply forget about it and say that I’m better now […] I have to take this seriously”* (P1) *“It’s an important question because it’s there to uncover a potential risk”* (P7) *“With that question, I’ve just ended up responding in the middle, because I’ve been thinking it over beforehand that ‘no… will it lead to anything?’”* (P8)
**3. Response basis variables**	
** 3.1. Degree of self-definition** This variable reflects that some phenomena only required the responder’s definition whereas others were more open to other people’s definitions and judgments. Typically, items concerning the responders’ thoughts, feelings, and symptoms are examples of private phenomena not visible to others and therefore easier to define and rate. In contrast, phenomena related to overt functioning would be more exposed to external judgment. Treatment and therapist-related items were also often difficult to outline for the responders without first being operationalized or more clearly defined within the treatment context	*“It’s just something I feel”* (P9) “*More like something I just know*” (P11) *“It’s not my area of expertise, and because of that I’m afraid to go into it and be the judge of something the other person clearly has the education and knowledge about, but I don’t”* (P1) *“I think maybe my treatment team might have defined something that I have not understood completely”* (P13)
** 3.2. Degree of context-dependence** This variable or dimension reflects that some phenomena would often change depending on the context. In the response process such phenomena were often indicated by the initial reaction ‘it depends on…’ and would require specification. For instance, items addressing social behaviors were often context-dependent and would often entail dilemmas for the responder, requiring them to specify what to focus on or to adapt their responses	*“This one is difficult, I think. Because it depends on who we’re talking about”* (P7) *“What kind of situations are we talking about?”* (P12) *“But it’s so dependent of things like..”* (P1) *“It’s not specific, because there’s so much in the background”* (P3)
** 3.3. Variability** This variable or dimension focuses on how the different response base phenomena change over time. Some phenomena, such as external resources, tended to stay stable across time and situations. Other phenomena change more frequently, from week to week, day to day, or even from one hour to the next. The response process phenomena that varied a lot would typically require more reasoning than stable phenomena	*“It changes from day to day, from the beginning of the week and towards the end. I could have answered a two, a four, a seven”* (P10) *“I think that one has changed a lot since the start of treatment”* (P4) *“The fluctuations are too large; it encompasses too much”* (P5) *“It’s one of those items where it is easy to think that ‘no, this has not changed since last time’”* (P2)
** 3.4. Need for justification** This variable reflects that some items contained assertions that needed to be justified. The participants would then search for “evidence” to support the claim. The evidence was typically autobiographical memories of events, but the justification could also be based on intentions or more general attitudes. An interesting aspect was that the types of evidence tended to have different weights; evidence including the responder’s actions would typically be valued more than justification based on thoughts or intentions	*“So I’m sitting there thinking about it and then the things that have improved come to mind”* (P3) *“I have to think about why… if I have to answer that I feel depressed most of the time, then I need to try to think it over. What is the reason for that?”* (P4) *“This one has annoyed me quite a lot, especially the ‘now I understand’ part of it. Sure, I can convince myself… I’ve felt it’s like a trap question. Sure, I could boost myself up, but do I really understand anything at all, right? So I’ve had to ask myself, ‘What is it that I understand?’”* (P2) *“I really should get through one year. Try to celebrate a birthday, some lows, see how it goes when other people are having a drink”* (P1)
** 3.5. Coping** Many of the items referred to problematic issues or symptoms, and this variable focused on how the respondents felt that they had been able to cope with the problem. Typically, this coping aspect became more relevant with repeated measures and a bit into the treatment course	“*I feel it all the time, but at times it is easier to distract yourself*” *(P5)* *“We’ve worked quite a lot with that, so I know some of the things that I have to do to get better”* (P4) *“I can feel uncomfortable in a setting with others, but still, I don’t stay at home because of it”* (P3) *“I can struggle, but if I am good at doing other things it’s not so bad. It’s only when… you see, it contributes to better measurement”* (P5)
** 3.6. Degree of certainty** The response base was associated with different degrees of certainty because of the variability, ambiguity, and shortcomings related to operationalization and justification. Increased certainty was typically related to past specific events, for instance, a recent example of coping, whereas less self-defined and context-dependent phenomena would often be associated with reduced certainty	*“I think five would be the correct answer still. Because there is still so much that is unknown*”* (P3*) *“I am uncertain about the future […] So, I am both positive and optimistic, but also very nervous and very uncertain at the same time. So, the answer is three-four”* (P8) *“It is often episodes, particularly negative events, that trigger. So I don’t know if I trust myself yet”* (P13) *I notice that I need to think a little. First, I think six. But then I have to think about that too. Because I need to see how things turn out in the time to come”* (P3)
**4. Reasoning strategies**	
** 4.1. Specifying** This variable refers to strategies that were used to specify the response base. This strategy was triggered when it was unclear what phenomena the items referred to and how they should be interpreted. Especially, with context-dependent phenomena, there would be several possible grounds and the responders sometimes had to decide what to emphasize. The need for specification would also increase with increased phenomena variability	*“I need to think first… kind of a question of definition” (P8)* *“Then think that I am often…”* (P12) *“How can that be measured?”* P5 *“I think it is about setting boundaries for yourself. At least where I am right now”* (P1)
** 4.2. Justifying** For assertions that might be judged differently by others the responders often tried to strengthen their reasoning by assembling proofs. As mentioned, evidence had different weights, where especially actions are valued. Consequently, having a recent event illustrating their point could have a major impact on the response. Examples representing evidence could both emerge spontaneously as associations or result from more effortful retrieval	*“To avoid that hesitation, I have to try to find arguments”* (P2) *“I know what I have to improve to recover and be well. So yeah… difficult question. I know in theory, but it is not that easy in practice”* (P7) *“I’ve tested going to parties and pubs […] so I come up with examples there”* (P1) *“People have started to trust me. It’s seven, it’s fine”* (P9)
** 4.3. Comparing** This variable refers to the fact that participants sometimes compared themselves against their past, other people, or some kind of perceived standard or ideal to create a reference frame for their answers. With repeated measures the findings suggest that participants typically would compare themselves with others initially but with repeated measures, gradually becoming more focused on comparing their present state to their previous, cf. the coping variable above	*“…after a while, you start to compare your own life against others’. Where we actually should be equal, but then it would be like you took on a role as someone who is ‘better’. For instance, it may be that I rated two, whereas for others it could be six, but still mean the same. But I try to answer based on what I feel in the moment, not necessarily compared to what others would have responded, although that’s probably some of what’s there in the background” (P13)* *“I think I should have managed much more without the therapist. That is the first thing I think about when I see this question”* (P2) *“When I’m responding to that question, I have to compare myself with others”* (P8) *“I have been thinking like ‘oh shoot what was it that I answered last time’. And think like ‘what if I score it lower now?’”* (P3)
** 4.4. Establishing a timeframe** This variable concerns the timeframe that the participants set for their response base. All items were preceded by the sentence ‘during the last week’ however it varied whether the participants took notice of this, and they would often include a longer period. A stricter specification of timeframe would, with repeated measures, often allow for more test score variability	*“There’s many of those [items] where you think that the question is actually bigger than only the last week”* (P11) *“I probably answered based on a longer timeframe”* (P3) *“‘The last week’ is a bit difficult, I think. Because the last week might have been fine, but in between there may have been things”* (P4) *“It’s two and a half or almost three on that one. But if you define the last week… then it is one”* (P13)
** 4.5. Generalizing** This variable focuses on to which degree the participants based their response on some general pre-established self-beliefs or what they perceived as their general patterns, rather than to evaluate specific recent events. With repeated measures generalizing would tend to flatten the score	*“For me, it is not based on the last week, but all weeks I would say*” (P6) *“…but that is more in general”* (P7) *“I just think about how it is in general”* (P12) *“I respond quickly because I think it has been like that the whole time and has not changed much for me”* (P4)
**5. Response adaptation strategies**	
** 6.1. Establishing a reference area** This variable entailed that the participants quickly established a reference area, usually encompassing two or three scores. A coarser estimate was to first decide whether they would be above or below the midpoint. Another aspect that occasionally needed to be considered was the direction of the scale, as the meaning of it would change depending on how the item was formulated. Establishing a reference area was a frequently used strategy, especially with repeated measures. It was also effective because it often meant that the responder could move from an item to the response adaptation process directly	*“There I am at a five, I might go up to a six, too”* (P3) *“It has always been above four”* (P7) *“The only thing I can do with such a question is really to put it on five or six because that way I’ve sort of moved above the midline”* (P8) *“Then I think that I’d really like to say seven”* (P13)
** 6.2. Adjusting** Having established a reference area, the responders often adjusted their scores before providing a numerical rating. The participants reported several different reasons for making such adjustments. For instance, they could adjust to mark improvement. However, they could also hold back to avoid moving too far in any direction, which might send out wrong signals or messages, for instance, that this was no longer a problem when they still felt they had a lot to work on. In consequence, the adjustments made were often only minor changes because the reasoning included counteracting considerations	*“I want to answer seven, but my thinking adjusts it down a little and that means that I will probably end up on five, because of the drugs and knowing that I can do better”* (P13) *“Now, I’ll adjust up, and that is because of my actions […] they indicate a seven”* (P2) *“…first four, then up on a five. But I think it will take a long time for me to get up to six or seven”* (P3) *“The first thing I’m thinking is that I’ll answer two, and then I think that perhaps I have improved, so I’ll respond three or four”* (P1)
** 6.3. Compromising** Compromising refers to the situations where the participants placed their score in the middle to encompass different aspects of the response base phenomena. This was a relatively frequently used strategy for some and typically related to responses regarding context-dependent phenomena or phenomena with high variability	*“I don’t know…it’s both-and. I would place myself in the middle there”* P12 *“So here I will respond in the middle each time. Because it depends on who”* (P7) *“This week I had both and I´m… It’s the kind of question that’s so open that I worry about moving too far in either direction, so I’ll answer four”* (P1) *“I end up lying anyway, I would say”* (P5)
**7. Response categories**	
** 7.1. Default responses** Default responses consisted of responses that the responders chose because they did not have a sufficient basis for their response. Such responses were typically related to items with poor clarity, where attempts to specify had been unhelpful. The score would typically be placed in the middle of the 1–7 Likert scale. Default responses could benefit from the item being clarified or from its meaning being more clearly operationalized in clinical conversations	*“.. it’s not based on much; I just think it’s above the middle”* (P8) *“The first time I got those questions I didn’t understand. I just answered four because I don’t like to be negative. Back then it was very much like that, I started at neutral”* (P3) *“If there had been some explanation of techniques and exercises the therapists had used it would have been more relevant. But as it is I don’t have any reference to what techniques and exercises mean”* (P13) *“I just answer something to get through it”* (P10)
** 7.2. Generalized responses** Generalized responses were outcomes of a generalized response base. Compared to default responses participants did have a response base; however, in the reasoning process, the responder would base the response on general impressions and without considering specific or recent events. Like the default responses, they tended to end up in the middle, perhaps with a tendency towards positive bias	“*I notice that I answer four more often than I really… that is, generally I have seen the question in a longer time perspective than I should have”* (P1) *“And now it’s been pretty much the same questions the last year and a half, so you hardly even have read the question”* (P4) *“It’s just based on intuition really; I remember how the sessions with the therapist usually are and that I kind of think they understand me ok”* (P8) *“I have to think back. And while I do that… I don’t know how relevant it’ll be for the therapist in the session that I start talking about last year”* (P10)
** 7.3. Adjusted responses** Adjusted responses consisted of responses that were a result of a preceding adjustment. Although there could be considerable reasoning behind the adjustments, they typically involved only minor changes because the reasoning tended to push the rating towards both lower and higher scores	*“That one is a good example where I could have answered two, then stopped and said to myself: the last week, this week – then gone back and answered three. Because each time there are one or two questions where I move to the next, and then go back again and change the answer. Not far, but one point”* (P1) *“I feel that… I’m worried that they will think I’m exaggerating if I respond that there’s much distress”* (P5) *“I could have answered four or five just to make a point. But I know that gut-anxiety-thing is sort of implied and that they already know about it. So I don’t need to score it that high”* (P8) *“A sense of shame tells me that it is way too early to answer six or seven”* (P1)
** 7.4. Compromises** Responses that reflected compromises were likely to be in the middle because they reflect the responder’s attempt to encompass different concerns and often represent an average of different possible responses	*“It had gone from negative to positive, but then I had to answer based on the last week, so I had to include both the positive and the negative”* (P7) *“P: On this one, the answer is one, four, six and seven. But I have to give one answer* *I: How do you solve that?* *P: I answer four. It feels like I have no other option because the possible answers differ so much”* (P1) *“I would respond four on that one because I know some things, but not everything”* (P9)
** 7.5. Distorted responses** Distorted responses refer to responses where the respondent misjudged the direction of the scale, typically with negatively formulated items. Such responses tended to occur less frequently in the current dataset than many of the other responses, however, they resulted in responses deviating considerably from the responder’s intended score	“*The ones where you have to be like ‘hello, am I going this or that way?’”* (P10) *“I took for granted that it [the question] was about the relationship between me and my therapist. Me, I actually think they’re doing well. I think I’ve answered, ‘not relevant’ here”* (P6) *“But I don’t know quite which direction it’ll go”* (P4) *“Some of the questions are ok, but then there are certain questions I don’t quite understand, should I be on this or that end of the scale. So I made some blunders from time to time”* (P10)
** 7.6. Communicative responses** Communicative responses include responses that were influenced by the responders trying to convey some message to the recipient. For example, giving higher or lower numerical ratings on items concerning issues they wanted to be addressed in the following session	*“But it is an important question, but also a bit like, if you respond anything above two on that one, it’ll be really important for the patient that the therapist addresses it properly”* (P8) “*It was more like a call for help, to put it like that. That this was something we had to talk about*” (*P7)* *“I feel that it is a way to signal what I want the upcoming session to be like, what I want to talk about, kind of”* (P4) *“I think I might be holding back and perhaps rate myself a little lower than what I feel to be true because I’m afraid of ending treatment too soon”* (P1)
** 7.7. Deficient responses** Deficient responses were related to the responder’s experienced quality of their response, which for some responders was characterized by a feeling of deficiency because they felt a need to add information and nuances, to their numerical ratings	*“I think so much more needs to be said about it. It’s not a yes or no question. […] There’s no answer that would fit me. The answer doesn’t fit.* (P6) *“I think it’s such a big question that I would have wanted to… it’s one of those questions where I would have liked to talk to someone for two hours”* (P1) *“.. if you had some extra alternative below, where you could explain more. Why you couldn’t answer. Because it isn’t that it’s not relevant. And it’s not that don’t want to answer, but I don’t have… I’m out of options”* (P5)
** 7.8. Non-responses** Non-responses were when the responders stopped answering. The participant mentioned several reasons why they either had stopped responding or considered doing so. A frequent complaint was the experience that nothing came out of it. Some reported that they had either stopped responding or responded only sporadically because of the experience that it was not followed up. It could also be related to item difficulty	“*It was questions like that, where it was so difficult to answer, that led to […] ‘I won’t bother to do this anymore’*” (*P5)* *“I stopped responding during outpatient treatment because it was never used. I felt that I couldn’t muster the energy to sit and answer because it demands quite a lot from you. The questions are personal and trigger thoughts and emotions and those kinds of things… so … when it was never used, it felt pretty meaningless”* (P11) *“I would probably not have continued responding if it was never mentioned or just been commented on now and then. That would have made it more difficult to motivate yourself”* (P4) * “Because many of the questions are very good and it’s a bit like… one could easily imagine ways in which the treatment and those questions could be linked in a good way. But then you might come to the session after having responded several times, and see the therapist just quickly go through it, and ask only a few questions, and that’s not very interesting. So that’s when you stop responding”* (P8)

### Context-related variables

Certain context-related variables were particularly important for how the participants experienced the purpose of the response process.

The participants reported different perceptions of **for whom and why** they were responding. Most imagined that they were responding to their therapist(s), but it could also be vague who the recipient was, and some felt like they were responding mostly for their own part. “*I’ve used this measure to think about how I am doing myself, more than to think that it will be used or read in the treatment”* (P1). It also varied between the participants how they experienced the **integration of the self-report measure in treatment**. While some felt that it was used very actively, others experienced little connection between the response process and the treatment process. “*I don’t know how relevant it feels in treatment, not so far at least. It’s not like anything they’ve asked about*” (P6).

### Item-related variables

The participants needed the items to make clear sense to initiate a meaningful response process. Their initial reactions encountering the different items suggest that certain properties of the items influenced the item’s impact and what kind of reasoning that were initiated.

Items differed in their degree of **clarity,** i.e. what specific phenomenon the item referred to or how it should be interpreted, e**.**g. “*It is not clear what they want to know from me*” (P10) or “*What is the definition of…”* (P13)*.* Items also differed in the degree of **relevance and utility** for the individual participants, e.g. “*That is not relevant for me*” (P6) or *“It’s like one of those core issues” (P13).*

Together, the context- and item-related variables influenced the participant’s motivation to respond and how much time they would spend on each item, e.g. “*It’s an important question because it activates me, it gets me thinking*” (P2), *“I respond quickly on that question because I get a bit overwhelmed by the size of it”* (P1), or “*There is nothing more to think about*” (P4). Thus, the items also elicited different reasoning pathways. For some items, the participants seemed to immediately have a tentative answer ready. For other items, they hesitated, became silent for a while, or reiterated part of the item, and reported thoughts that indicated more elaborative reasoning to generate a response base.

### Response base variables

To provide a meaningful response the participant needed a response base, i.e. a conceptual foundation on which to base the response. The following aspects related to the response base were identified as influential to what reasoning strategies the participants utilized to provide their responses.

First, phenomena that constituted potential response bases varied in the degree of **self-definition**. Some phenomena only required the responder’s own definition, *“It’s just something I feel”* (P9) or “*More like something I just know*” (P11)*.* Other phenomena were more susceptible to other people’s definitions and judgments, e.g. *“It’s not my area of expertise”* (P1). **Context-dependent** phenomena were typically indicated by the initial reaction “*it depends on…*” and often entailed several possible interpretations: *“What kind of situation are we talking about?”* (P12). A third aspect of the response base influential to the reasoning process was the **variability** of the phenomena in question, *“It changes from day to day” (P10)* or “*It doesn’t change, so it’s kind of nothing more to think about*” (P4). The response base also varied regarding the **need for justification***.* Whereas self-defined and decontextualized phenomena often were self-evident, phenomena exposed to other’s judgment needed to be justified. *“So, I’ve had to ask myself ‘what is it that I understand?”* (P2)*.* With repeated measures, some participants also questioned whether to base their response according to frequency or presence of specific phenomena, or their experience of **coping** with the phenomenon in question. “*I feel it all the time, but at times it is easier to distract yourself*” *(P5)*. Consequently, the response base became associated with different degrees of **certainty** “*There is still so much that is unknown*” *(P3*) and required different reasoning strategies.

### Reasoning strategies

Due to the varying reasoning requirements of the different response base phenomena, the participants adopted reasoning strategies to improve the response base. The choices made and what examples happened to be available in their consciousness during the reasoning process could have a great impact on their numerical ratings.

With unclear, context-dependent, or high variability phenomena there would be several possible bases for a response and consequently, the participants had to engage in a process of **specification** to define what to focus on: *“I need to think first… kind of a question of definition” (P8).* For assertions that might be judged differently by others, the participants would often try to strengthen their response base by retrieving evidence to **justify** their response. *“To avoid hesitation, I have to find arguments” (P2).* The participants sometimes also made **comparisons** to create a reference frame. *“…then you start to compare your own life against others” (P13).* **Establishing a timeframe** could have a great impact on the response, especially in cases where the phenomena in question showed high variability. “*It varies from day to day, at the beginning of the week compared to the end, so I could have answered a two, a four, a seven.*” *(P10).* However, as the abovementioned strategies often required effort and entailed shortcomings, a typical solution was to **generalize**: *“I just think about how it is in general”* (*P12).*

### Response selection strategies

When the response base was considered sufficient or further reasoning unhelpful, response adaptation strategies were used for many of the items to translate the participant’s response into a numerical rating on the 1–7 Likert scale.

For many items the participants quickly **established a reference area**, usually encompassing two or three potential scores, e.g. “*It has always been above four” (P7)*. Before deciding on a specific numerical rating, however, the responders often **adjusted their score**, e.g. “*Now I adjust myself up, and that is because of my actions, that indicates a seven”* (P2). Another frequently used adaptation strategy was to **compromise** to encompass the different potential responses participants could give to phenomena that varied according to context or how they were specified. *“During this last week I’ve had both, and I become… it’s a kind of question that is so open that I become […] worried about moving too far in either direction, so I will answer four”* (P1)*.*

### Resulting response categories

Due to the influence of different response process variables, many of the numerical item responses could be categorized based on how they were affected by the different aspects of the response process.

A first cluster of response categories was primarily related to the response base and reasoning strategies. **Default responses** consisted of numerical ratings founded on an insufficient response base., e.g. “*It’s not based on much; I just think it is above the middle*” (P8). **Generalized responses**, on the other hand, were based on participants’ general perceptions of themselves, often with an associated numerical rating already stored in memory, i.e. not the result of any evaluation of current events or recent examples. “*I notice that I answer four more often than I really… that is, generally I have seen the question in a longer time perspective than I should have”* (P1).

A second cluster of the response categories was primarily related to the response selection process. **Adjusted responses** consisted of numerical ratings that resulted from a preceding adjustment. Although there could be considerable reasoning behind the adjustments, they typically involved only minor changes because the reasoning tended to push the rating towards both lower and higher scores. “*I want to answer seven, but my thinking adjusts it down a little and that means that I will probably end up on five, because of the drugs and knowing that I can do better”* (*P13).* Like adjusted responses, **compromises** tried to encompass different concerns, but were more likely to be in the middle of the scale and represent an average of different possible responses: *“It had gone from negative to positive, but then I had to answer based on last week, so I had to include both the positive and the negative”* (P7). **Distorted responses** consisted of numerical ratings where the respondent misjudged the direction of the scale, typically confusing higher scores reflecting a more positive outcome, when in fact the item is negatively formulated. “*The ones where you have to be like ‘hello, am I going this or that way?’”* (P9)*.*

A third cluster of response categories was related to context and item-related variables. **Communicative responses** were influenced by the responder wanting something to be addressed in the following session. “*It was more like a call for help, to put it like that. That this was something we had to talk about.*” (*P7).* **Deficient responses** were characterized by experienced communicative limitations of the response, typically related to its inability to convey additional nuances. “*I think so much more needs to be said about this*.” (*P6).* Finally, **non-responses** concern cases where the participant had stopped responding to the item because of the cost associated with the response process or the experience that little came out of it. “*It was questions like that, where it became so difficult to answer, that led to […] ‘I won’t bother to do this anymore’.*” (*P5).*

## Discussion

In this study, we investigated the response processes of patients providing quantitative self-report data. The aim was to identify factors that influence the response process and to develop a theory that can guide what to look for when considering validity and interpreting scores. We found that as the participants proceeded from reading the question to providing a numerical response, they were influenced by variables related to context, items, response base, reasoning process, and the process of adapting their response to a numerical score. We also found that many item responses could be categorized based on how they were affected by the different aspects of the response process. We refer to Table 2 for a more detailed description of the variables in the response process and the response categories.

The response categories may have important implications for how scores from quantitative self-report measures should be interpreted and followed up in this context. Clinical interpretations need to be verified for each specific patient and item, but based on the present results, we suggest that clinicians and researchers consider midpoint sometimes reflect default responses or compromises due to insufficient or conflicting response bases. In comparison, scores slightly above or below the middle score (i.e., 3 or 5) are more likely to reflect adjusted or communicative responses. With repeated measures, high variability on individual item responses may indicate specified response bases and established timeframes. Item responses with low variability may reflect stable phenomena, however, assessing the underlying response process may reveal generalized or deficient responses.

Previous research has also investigated how cognitive information processing influences responses but has typically focused on how the responder’s cognitive operations or item characteristics influence the accuracy of the response [13, 15]. In a clinical context and with self-evaluate items, however, participants are seeking to communicate with their provider. This may affect the response process in many ways. For instance, in two different cases where the patient had experienced a recent relapse of substance abuse, one of them experienced the response process as a burden and something to be finished as quickly as possible, whereas the other patient saw this as an opportunity to communicate important information to the therapist. While we cannot say for certain that the therapist in the former case should have done anything differently than they did, this finding coincides with a considerable literature e.g. [26–28] suggesting that therapists’ active use of measurements and explicit discussion of measurement during treatment strongly affects patients’ attitudes towards the measurement itself.

One way to conceptualize the relationship between response and treatment processes is that active use of the feedback system in treatment sessions allows for a shared reasoning process in which the meaning of items and responses can be elaborated and agreed upon in the session. Ideally, patients should trust that their therapists will discuss their responses on the measure in sessions, because their therapist will review the patients’ responses prior to clinical contacts. In-session discussion could reduce the frequency of default and generalized responses, explicate adjusted responses, identify communicative responses and concealed messages in deficient responses, and possibly prevent non-responses. Thoroughly integrating the measures into clinical care can thus partially compensate for shortcomings in the item response process and create a shared understanding of response meaning. Thus, even item responses and scores that have complex idiographic interpretations can be validly used in treatment through collaborative discussion.

To our mind, the main strength of this study is that it combines cognitive interviewing and grounded theory to generate knowledge about a subject area that has been insufficiently investigated in previous research. By taking the responders’ points of view we were able to identify the conditions and challenges that they consciously face in the response process. The reliance on the participants’ verbal self-report may, however, limit generalizability. The interview setting might also have influenced the apparent response process. In particular, the presence of an interviewer might have motivated elaboration and explanation, rather than a direct reflection of actual thought processes. Moreover, because we synthesized data across items and participants, there might be important variability across items and individuals that were not fully explored. The generalizability of the findings may be further enhanced by testing a broader range of items, and transferability should be tested by including other patient populations and settings.

## Conclusion

The main implication of this study is that the validity of inferences and actions based on quantitative self-report measures used in mental healthcare and substance use disorder treatment can be improved by considering response processes. The proposed model may be well suited to suggest how measurements can be adapted, for instance, to guide the development and revision of individual items. The response process variables and response categories also have implications for how ROM/CFS self-report measures should be interpreted during treatment: notably, that the total score might belie complex and communicative underlying processes, variable across each patient and timepoint. This should affect how therapists use the item responses for treatment modification.

## Supplementary Information

Below is the link to the electronic supplementary material.

Supplementary file1 (PDF 266 kb)

Supplementary file1 (PDF 179 kb)

## Data Availability

Data is qualitative interviews in Norwegian and can be made anonymously and available on reasonable request within 5 years of publication data.

## References

[1] Hawkins, M., Elsworth, G. R., & Osborne, R. H. (2018). Application of validity theory and methodology to patient-reported outcome measures (PROMs): Building an argument for validity. *Quality of Life Research,**27*(7), 1695–1710. 29464456 10.1007/s11136-018-1815-6 PMC5997725

[2] Zumbo, B. D., & Chan, E. K. H. (2014). Validity and validation in social, behavioral, and health sciences. *Springer International Publishing*. 10.1007/978-3-319-07794-9

[3] American Educational Research Association, American Psychological Association, & National Council on Measurement in Education (Eds.). (2014). *Standards for educational and psychological testing*. Americal Educational Research Association.

[4] Hubley, A. M., & Zumbo, B. D. (2017). Response processes in the context of validity: Setting the stage. In B. D. Zumbo & A. M. Hubley (Eds.), *Understanding and investigating response processes in validation research* (pp. 1–12). Springer International Publishing.

[5] De Jong, K., Conijin, J. M., Gallagher, R. A. V., Reshetnikova, A. S., Heij, M., & Lutz, M. C. (2021). Using progress feedback to improve outcomes and reduce drop-out, treatment duration, and deterioration: A multilevel meta-analysis. *Clinical Psychology Review,**85*, 102002. 10.1016/j.cpr.2021.102002 33721605 10.1016/j.cpr.2021.102002

[6] Barkham, M., De Jong, K., Delgadillo, J., & Lutz, W. (2023). Routine outcome monitoring (ROM) and feedback: Research review and recommendations. *Psychotherapy Research,**33*(7), 841–855. 10.1080/10503307.2023.2181114 36931228 10.1080/10503307.2023.2181114

[7] Låver, J., McAleavey, A., Valaker, I., Castonguay, L. G., & Moltu, C. (2023). Therapists’ and patients’ experiences of using patients’ self-reported data in ongoing psychotherapy processes—a systematic review and meta-analysis of qualitative studies. *Psychotherapy Research*. 10.1080/10503307.2023.2222896 37322037 10.1080/10503307.2023.2222896

[8] Launeanu, M., & Hubley, A. M. (2017). Some observations on response processes research and its future theoretical and methodological directions. In B. D. Zumbo & A. M. Hubley (Eds.), *Understanding and investigating response processes in validation research* (pp. 93–113). Springer International Publishing.

[9] Tourangeau, R. (1984). Cognitive Sciences and survey methods: A cognitive perspective. In T. Jabine, M. Straf, J. Tabur, & R. Tourangeau (Eds.), *Cognitive aspects of survey design: Building a bridge between disciplines* (pp. 73–100). National Academy Press.

[10] Schwarz, N., & Oyserman, D. (2001). Asking questions about behavior: Cognition, communication, and questionnaire construction. *American Journal of Evaluation,**22*(2), 127–160. 10.1016/S1098-2140(01)00133-3

[11] Jobe, J. B. (2003). Cognitive psychology and self-reports: Models and methods. *Quality of life research,**12*(3), 219–227. 10.1023/a:1023279029852 12769134 10.1023/a:1023279029852

[12] Schwarz, N. (2007). Cognitive aspects of survey methodology. *Applied Cognitive Psychology,**21*(2), 277–287. 10.1002/acp.1340

[13] Tourangeau, R. (2018). The survey response process from a cognitive viewpoint. *Quality Assurance in Education,**26*(2), 169–181. 10.1108/QAE-06-2017-0034

[14] Miller, K., & Willis, G. B. (2016). *The SAGE handbook of survey methodology*. SAGE Publications Ltd.

[15] Schwarz, N., Knäuper, B., Oserman, D., & Stich, C. (2012). The psychology of asking questions. *International Handbook of Survey Methodology*. 10.4324/9780203843123

[16] Nathaniel, A. (2023). The logic and language of classical grounded theory: Induction, abduction, and deduction. *Grounded Theory Review,**22*(1), 17–22.

[17] Willis, G. B. (2015). *Analysis of the cognitive interview in questionnaire design*. Oxford University Press.

[18] Beatty, P. C., & Willis, G. B. (2007). Research synthesis: The practice of cognitive interviewing. *Public Opinion Quarterly,**71*(2), 287–311.

[19] Charmaz, K. (2014). *Constructing grounded theory* (2nd ed.). Sage.

[20] Birks, M., & Mills, J. (2022). *Grounded theory: A practical guide*. Sage.

[21] Glaser, B., & Strauss, A. (2017). *Discovery of grounded theory: Strategies for qualitative research*. Routledge.

[22] Nordberg, S. S., McAleavey, A. A., & Moltu, C. (2021). Continuous quality improvement in measure development: Lessons from building a novel clinical feedback system. *Quality of Life Research,**30*(11), 3085–3096. 10.1007/s11136-021-02768-7 33591432 10.1007/s11136-021-02768-7 PMC8528746

[23] McAleavey, A. A., Nordberg, S. S., & Moltu, C. (2021). Initial quantitative development of the norse feedback system: A novel clinical feedback system for routine mental healthcare. *Quality of Life Research.,**30*(11), 3097–3115. 10.1007/s11136-021-02825-1 33851326 10.1007/s11136-021-02825-1 PMC8528796

[24] Levitt, H. M. (2021). *Essentials of critical-constructivist grounded theory research*. American Psychological Association.

[25] Malterud, K., Siersma, V. D., & Guassora, A. D. (2015). Sample size in qualitative interview studies: Guided by information power. *Qualitative Health Research,**26*(13), 1753–1760. 10.1177/1049732315617444 10.1177/1049732315617444 26613970

[26] Boswell, J. F., Kraus, D. R., Miller, S. D., & Lambert, M. J. (2015). Implementing routine outcome monitoring in clinical practice: Benefits, challenges, and solutions. *Psychother Research,**25*(1), 6–19. 10.1080/10503307.2013.817696 10.1080/10503307.2013.817696 23885809

[27] Unni, E., Coles, T., Lavalle, D. C., Freel, J., Roberts, N., & Absolom, K. (2024). Patient adherence to patient-reported outcome measure (PROM) completion in clinical care: Current understanding and future recommendations. *Quality of Life Research,**33*(1), 281–290. 10.1007/s11136-023-03505-y 37695476 10.1007/s11136-023-03505-y PMC10784330

[28] Solstad, S. M., Kleiven, G. S., & Moltu, C. (2021). Complexity and potentials of clinical feedback in mental health: An in-depth study of patient processes. *Quality of Life Research,**30*(11), 3117–3125. 10.1007/s11136-020-02550-1 32556824 10.1007/s11136-020-02550-1 PMC8528773

