# Supplementary material for: Response processes for patients providing quantitative self-report data: a qualitative study
Source: Qual Life Res. 2024 Aug 14;33(11):2949–61. doi: 10.1007/s11136-024-03749-2 (PMC11541247; doi:10.1007/s11136-024-03749-2)
Supplement: Supplementary file 1 — Supplementary file1 (PDF 266 kb) [file 11136_2024_3749_MOESM1_ESM.pdf]

Interview guide (Inspired by: Willis, G. B. (2004). *Cognitive Interviewing: A tool for improving questionnaire design*. Sage Publications.)

General introduction to the think aloud condition:

You will now be presented with items from the Norse Feedback. What I want you to do first, is to respond to these items as you would have if you have encountered the same items as part of your treatment. However, this time I ask you to also try to describe your thoughts while responding.

Questions asked when appropriate to encourage the participants to continue to think aloud:

What did you start to think about?
How would you describe your thought process while you were answering this item?

Probing questions (asked when something needed to be investigated further or the think aloud condition gave little information)**:**
From your perspective, what is this question about?
What do you think about when responding to that item?
What does … mean to you?
Do you experience any emotional reaction to this item?
Are you experiencing any challenges when responding to this item?
What makes this item easy/difficult to understand?
Can you repeat that question in your own words?
What do you base your answer on?
Did you have an answer ready straight away, or did you need to think about it?
Where on the scale would you place your answer?
What influenced your response?
What would it take to score it higher or lower?
Were you able to find an appropriate answer?
Was there anything you would have said instead?
When you respond to that item, is there something you hope will happen next?
Has there been a change in the issue this item covers?

Questions about experiences of using Norse Feedback (NF) in treatment (asked at the end of the interview):
What are your experiences of using NF in treatment?
Have there been any specific episodes where it has been particularly helpful or challenging?
Have any of the items been particularly helpful or challenging?
Is there anything you miss being asked about?
If you could make changes to the feedback system, what would they be?
