# Supplementary material for: Response processes for patients providing quantitative self-report data: a qualitative study
Source: Qual Life Res. 2024 Aug 14;33(11):2949–61. doi: 10.1007/s11136-024-03749-2 (PMC11541247; doi:10.1007/s11136-024-03749-2)
Supplement: Supplementary file 2 — Supplementary file1 (PDF 179 kb) [file 11136_2024_3749_MOESM2_ESM.pdf]

Additional information about the recruitment process

The recruitment period lasted from March to December 2023. Participants were recruited from a specialized substance use disorder treatment clinic located in the central part of Norway. We recruited participants who were in different phases of treatment and had different experiences with using feedback measures in treatment. It was not required that the participants had used the Norse Feedback (NF) system in their treatment themselves, but we recruited participants from treatment units that had implemented the NF system. This excluded participants from a detoxification unit and drug-assisted rehabilitation unit at the same clinic. Apart from that there were no exclusion criteria. To ensure that the sample reflected a wide range of the patient population, we recruited participants from both outpatient (n=10) and inpatient (n=3) treatment settings. Eligible outpatient participants were identified based on an earlier consent to participate in another study related to the same research project. In the consent form they had been informed that they might be contacted in connection with this study. Eligible participants were sent a SMS asking if they were interested in receiving a telephone call with further information about the project. In the SMS they were told that they could respond *no* if they did not want to be contacted. Those who did not decline were called, provided detailed information about the study, and then given a few days to consider if they wanted to participate or not. For those interested in participating an interview was scheduled. Altogether, n = 31 were contacted via SMS. Seven declined via SMS, ten did not respond neither to the text message nor later phone call, and four were not able to participate for other reasons (did not have time, did not attend scheduled appointment, had not recently been in SUD treatment). We recruited ten participants via this approach. In addition, we recruited three participants from an inpatient unit at the same clinic. For the inpatient recruitment, information of the study was first presented in a joint patient meeting at the unit. Potential patients attending the meeting was then given a few days to consider whether they were interested. Those who decided that they were interested, could sign up on a list made available. The first author contacted those who had signed up one by one and gave a more thorough information and scheduled interviews for those who wanted to participate. Approximately twelve patients attended the first information meeting and five of them signed up on the list provided. Of these five potential participants, two could not participate due to changes in their treatment. Three interviews were conducted based on participants from the inpatient unit. There was no systematic difference between outpatient and inpatient participants in the study other than their current level of care.

We wanted the participants to be in different stages of treatment and have different level of experience with using feedback systems in treatment (including the NF system). Therefore, we continued to recruit participants until we had sufficient variation in our sample. The last half of the recruitment process was done sequentially to allow for simultaneous data collection and analysis. To evaluate the needed sample size new incoming data were continuously compared to existing data. We stopped recruiting new participants when the last three interviews did not add any significant new information compared to existing data.
